# Case Report: Concomitant BRAF V600E and PIK3CA H1047R mutations in a micro-papillary thyroid carcinoma with extensive nodal metastasis

**DOI:** 10.3389/fonc.2026.1887104

**Published:** 2026-08-04

**Authors:** Linkun Zhong, Zhibin Huang, Xingyu Song, Yutong Li, Dean Wu

**Affiliations:** 1 Department of General Surgery, The Third People’s Hospital of Gansu Province, Lanzhou, Gansu, China; 2 The Department of General Surgery, Zhongshan City People’s Hospital, Zhongshan, Guangdong, China; 3 Medical School, Shenzhen University, Shenzhen, Guangdong, China; 4 The First School of Clinical Medicine, Guangdong Medical University, Zhanjiang, Guangdong, China

**Keywords:** BRAF V600E, case report, lymphatic metastasis, molecular profiling, papillary thyroid carcinoma (PTC), papillary thyroid microcarcinoma (PTMC), PIK3CA H1047R

## Abstract

Although most papillary thyroid carcinoma (PTC) cases follow an indolent clinical course, a small subset presents with early extensive lymph node metastasis. Although this aggressive phenotype is frequently linked to the BRAF V600E mutation, concurrent activating mutations in the PI3K/AKT pathway, such as PIK3CA H1047R, are exceedingly rare and their clinical significance remains poorly understood. We report the case of a 42-year-old male with a 7.8-mm solitary papillary thyroid microcarcinoma (PTMC) harboring concurrent BRAF V600E and PIK3CA H1047R mutations. Despite the small size of the primary lesion, extensive cervical lymph node metastases were identified in at least five nodal levels. Although the primary tumor was classified as a low-risk by conventional size-based criteria, the presence of these two oncogenic drivers suggests disproportionately aggressive lymph node dissemination relative to tumor size, underscoring the value of expanded molecular profiling for risk stratification.

## Introduction

Papillary thyroid carcinoma (PTC) is the most common malignant of the thyroid gland. Although most cases PTCs follow an indolent course, a small subset exhibits aggressive behavior with early lymph node metastasis (1). In the majority of PTC cases, the BRAF V600E mutation is associated with extrathyroidal extension and lymph node metastasis (2, 3). However, concurrent BRAF V600E and PIK3CA H1047R mutations are detectable in fewer than 5% of PTC cases, and the clinicopathological significance of this co-occurrence remains poorly understood (4, 5). Papillary thyroid microcarcinoma(PTMC) is defined as a thyroid papillary tumor with a diameter of ≤ 10 mm, and generally carries a favorable prognosis and a very low risk of metastasis (6). PTMC with concurrent BRAF V600E and PIK3CA H1047R mutations and extensive cervical lymph node metastasis is extremely rare. Here, we report this rare case to highlight the potential value of expanded molecular profiling in risk stratification for PTMC, and to discuss the implications of concomitant BRAF V600E and PIK3CA H1047R mutations for clinical decision-making in patients with small-volume, high-risk disease.

## Case presentation

A 42-year-old male presented with a 2-year history of a right cervical mass. He reported no associated symptoms, including local pain, fever, palpitations, or weight loss. There was no personal or family history of thyroid disease or other malignancies. Apart from a palpable, firm, non-tender mass in the right neck, no abnormalities were found in the physical examination.

**Figure 1:**
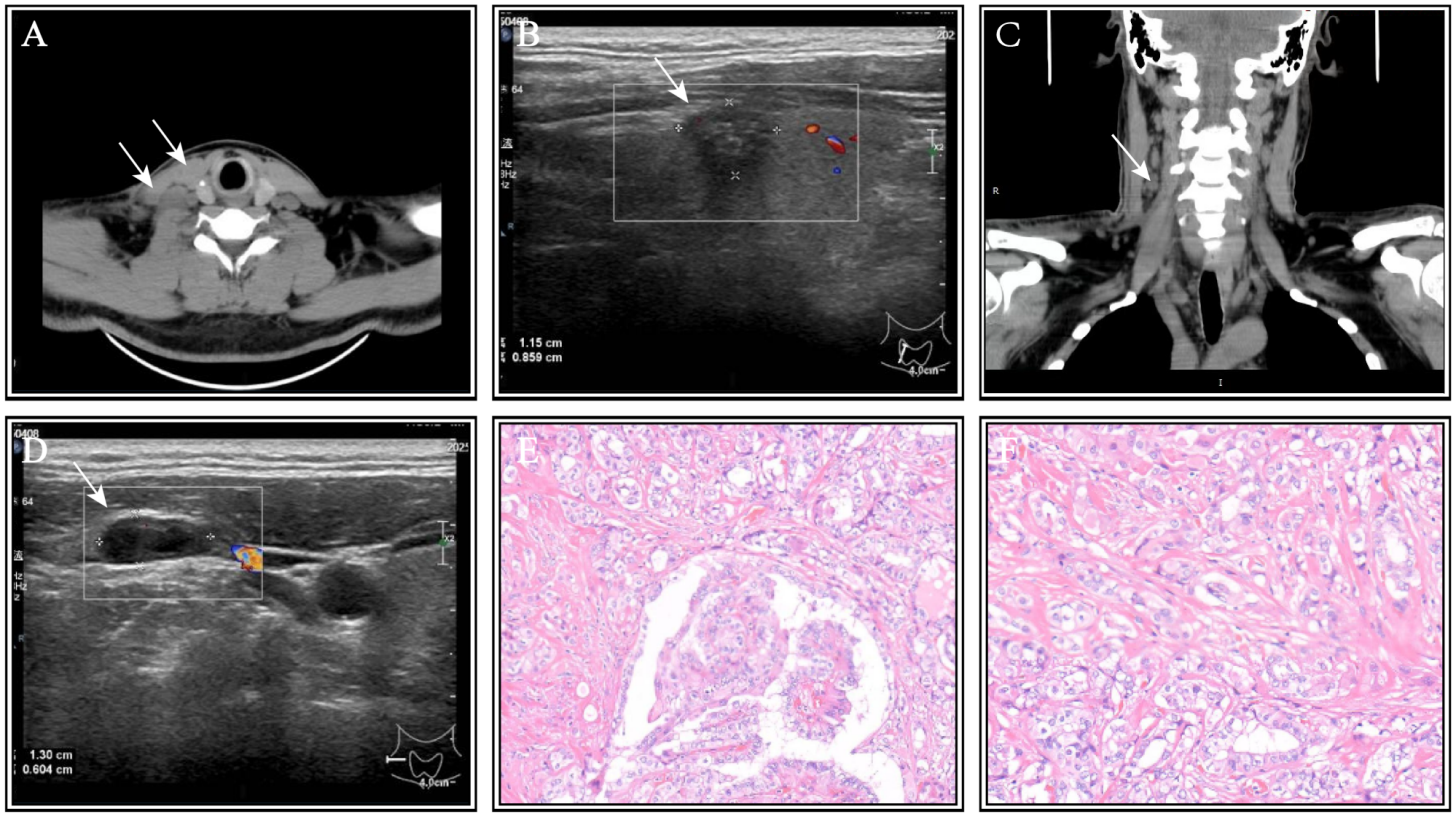
Imaging and pathological findings of the case. **(A)** Contrast-enhanced CT of the neck shows multiple indeterminate lesions in the right supraclavicular fossa. **(B)** The thyroid ultrasound reveals a hypoechoic nodule (11 × 9 mm) in the right thyroid lobe with microcalcifications and possible capsular discontinuity. **(C)** CT of the neck demonstrates multiple enlarged lymph nodes in the right levels III, IV, and VI. **(D)** thyroid ultrasound section show right supraclavicular lymphadenopathy. **(E)** Histopathology of the primary tumor (hematoxylin and eosin, ×100) shows classic papillary architecture with fibrovascular cores and ground-glass nuclei. **(F)** Histopathology of a metastatic lymph node (hematoxylin and eosin, ×100) confirms PTC metastasis with similar nuclear features.

Contrast-enhanced computed tomography (CT) of the neck revealed multiple indeterminate lesions in the right supraclavicular fossa (**Figure 1A**). The thyroid ultrasound examination identified a 11 × 9 millimeter hypoechoic nodule in the right thyroid lobe, accompanied by microcalcification and possible discontinuity of the capsule (**Figure 1B**). Moreover, multiple abnormal lymph nodes were found in the right cervical levels III, IV, and VI as well as the right supraclavicular region (**Figures 1C, D**).

In March 2025, the patient underwent a resection biopsy of a single right cervical lymph node at the referring hospital. This was an excisional biopsy, not a formal neck dissection. Histopathological examination confirmed metastatic PTC in the lymph nodes, while a separately sampled right level V lymph node showed no malignancy. The patient subsequently underwent total thyroidectomy with right central compartment neck dissection and right modified radical neck dissection. No significant abnormalities were identified, and the postoperative course was uneventful.

Histopathological examination of the surgical specimen confirmed a solitary PTC lesion in the right thyroid lobe, measuring 7.81 mm in maximum diameter. The tumor demonstrated capsular invasion without vascular invasion, corresponding to the pathological stage of pT1a (**Figures 1E, F**). No tumor was identified in the left thyroid lobe or isthmus. Metastatic PTC was identified at multiple nodal sites: right level II (1/10), right level III (2/11), right level IV (1/1), right level VI B (2/6), and the central compartment (1/1).

Immunohistochemically, the tumor cells were positive for CK19 and BRAF mutant protein (VE1) and negative for thyroid peroxidase (TPO). Postoperative molecular testing was performed using fluorescence-based polymerase chain reaction (PCR) on DNA extracted from formalin-fixed paraffin-embedded tumor tissue. The analysis confirmed the presence of the BRAF V600E (c.1799T>A) and PIK3CA H1047R (c.3140A>G) mutations in the tumor specimen.

The patient recovered uneventfully and was discharged on postoperative day 3. He did not receive radioiodine ablation or other adjuvant therapy. Follow-up neck ultrasound performed at 4 and 7 months after surgery revealed no evidence of residual or recurrent disease. The patient remains under regular monitoring. The chronological sequence of key events is summarized in **Table 1**.

**Table 1:** Timeline of the clinical course.

Date	Event
2023 (approximate)	Patient first noticed a right neck mass
March 2025	Initial presentation; contrast-enhanced CT and thyroid ultrasound performed
March 2025	Excisional biopsy of right neck mass confirmed metastatic PTC
April 2025	Total thyroidectomy + right central neck dissection + right modified radical neck dissection
April 2025	Histopathology confirmed 7.81 mm PTMC, pT1a, with multilevel nodal metastases
April 2025	Immunohistochemistry: CK19+, BRAF VE1+, TPO−
May 2025	Next-generation sequencing confirmed BRAF V600E and PIK3CA H1047R mutations
August 2025	4-month follow-up: neck ultrasound showed no recurrence
November 2025	7-month follow-up: neck ultrasound showed no recurrence

The timeline illustrates the chronological sequence of key events from initial presentation through postoperative follow-up in a 42-year-old male with BRAF V600E/PIK3CA H1047R co-mutated papillary thyroid microcarcinoma.

## Timeline

The timeline illustrates the chronological sequence of key events from initial presentation through postoperative follow-up in a 42-year-old male with BRAF V600E/PIK3CA H1047R co-mutated papillary thyroid microcarcinoma.

## Discussion

This case describes a 7.8-mm papillary thyroid microcarcinoma (PTMC) that harboring concurrent BRAF V600E and PIK3CA H1047R mutations, which presented with lymph node metastases across at least five cervical levels. The co-occurrence of these two mutations offers a plausible explanation for the aggressive phenotype. BRAF V600E and PIK3CA H1047R respectively activate the MAPK pathway and the PI3K/AKT/mTOR pathway, and the co-activation of these two cascades is frequently associated with aggressive thyroid cancer progression. In a recent genomic profiling study of anaplastic thyroid cancer, PI3K/Akt/mTOR pathway variants, including PIK3CA, were identified in 26.6% of cases, suggesting that these variants are prevalent in the most aggressive thyroid malignancies (7). Another preclinical study showed that combined MAPK and PI3K inhibition reversed BRAF V600E-driven dedifferentiation and restored follicular organization in thyroid cancer organoids (8). These findings support the notion that dual mutations contribute to a metastatic phenotype and enhanced aggressive progression.

A 2025 systematic review and meta-analysis reported that PI3K pathway mutations were associated with significantly shorter overall survival (9). Furthermore, mutations in PIK3CA have been associated with resistance to BRAF V600E inhibitors and aggressive progression toward anaplastic histology (10). Previous studies have shown that BRAF V600E mutation is associated with lymph node metastasis and extrathyroidal invasion in PTC and PTMC (11). Our case extends these observations by suggesting that concurrent BRAF V600E and PIK3CA H1047R mutations may further enhance aggressive behavior, even in a microcarcinoma, supporting the potential value of preoperative molecular profiling in guiding surgical decision-making. Although outcome data on BRAF V600E/PIK3CA co-mutation in differentiated PTC remain limited, the available evidence provides a plausible explanation for the extensive lymph node spread observed in our patient.

PTMC is generally considered to carry a low risk of lymph node metastasis, with a lymph node metastasis rates of 14.5% and 15.9% in two recent cohort studies (6, 12). In addition, the 2025 Korean Thyroid Association guidelines suggest that active surveillance may be considered for low-risk PTMC without nodal or distant metastases, extensive extrathyroidal extension, or evidence of aggressive histology (13). Thus, by these criteria, this tumor are classified as low risk based on size alone. However, the primary tumor itself, at 7.8 mm, would have otherwise qualified for conservative management or even active surveillance based on size alone. This paradox highlights the critical disconnect between tumor size and its biological potential, and supports the emerging concept that molecular risk assessment should be integrated with conventional clinicopathological factors to guide personalized treatment strategies (14).

PIK3CA mutations also have therapeutic implications for thyroid cancer. In a 2025 review of the high-risk molecular features of radioiodine-refractory differentiated thyroid cancer, it was documented that BRAF V600E and promoter mutations drive MAPK pathway activation and lead to subsequent sodium-iodine symporter inhibition, while alterations in the PI3K/AKT pathway further contribute to the loss of iodine affinity (15). PIK3CA mutations are rare in differentiated thyroid cancers (approximately 1-2%) but are more frequent in aggressive subtypes, where they are associated with disease progression. The H1047R mutation constitutively activates the PI3K/AKT pathway, promoting cell survival, proliferation, and metastasis. Although no additional therapy is currently required, the molecular profile suggests a potential therapeutic role for combined targeted therapy should relapse occur and iodine avidity be lost.

This case report has several limitations. The seven-month follow-up period is insufficient to assess late relapse. As a single case, these findings are not generalizable. No comprehensive genomic analysis was performed for other mutations, such as TERT promoter or TP53 alterations. Despite these limitations, this case demonstrates shows that BRAF V600E/PIK3CA co-mutation can occur in PTMC with extensive lymph node spread. If expanded molecular testing identifies PTMC patients with high-risk genotypes, they may benefit from more individualized surgical planning and closer postoperative surveillance. This also underscores the need for larger studies with long-term follow-up to validate this approach.

## Conclusion

This case demonstrates that a subcentimeter papillary thyroid carcinoma harboring concurrent BRAF V600E and PIK3CA H1047R mutations can present with extensive cervical lymph node metastasis. The co-occurrence of these two mutations, which activate the MAPK and PI3K/AKT pathways respectively, provides a molecular explanation for the discrepancy between the small primary tumor size and the extensive nodal involvement. Our findings suggest that a hypothesis-generating observation is that expanded molecular profiling may identify a subset of PTMC patients with high-risk genotypes. This could potentially benefit from more extensive initial surgery and closer surveillance, but this hypothesis requires validation in larger, long-term follow-up studies before it can be translated into clinical practice. Larger studies with longer follow-up are needed to validate this approach and to define optimal management strategies for this molecularly defined high-risk subgroup.

## Patient perspective

The patient provided written informed consent for publication of this case report. He reported he first noticed a mass on the right side of his neck more than two years ago before presenting to the hospital. As the mass had caused no pain or discomfort, he postponed seeking evaluation. Upon learning that the biopsy showed metastatic thyroid cancer, he was surprised and scared. He was apprehensive when total thyroidectomy and extensive lymphadenectomy were recommended, but he was reassured after the team explained the preoperative evaluation. He reported an uneventful recovery and was relieved that he did not require radioiodine therapy or other adjuvant treatment. On a subsequent follow-up visit, he was rather reassured after learning that the ultrasound examination showed no evidence of tumor recurrence. He stated the experience taught him the importance of not neglecting further evaluation even in the absence of symptoms.

## Data Availability

The original contributions presented in the study are included in the article/supplementary material. Further inquiries can be directed to the corresponding authors.
